# Role of Social Stress on synaptic plasticity and mood disorders in Alzheimer's Disease. Mechanisms and Therapeutic Implications.

**DOI:** 10.1002/alz70859_107252

**Published:** 2025-12-26

**Authors:** Giulio Maria Pasinetti, Eun‐Jeong Yang, Nicole Less

**Affiliations:** ^1^ Icahn School of Medicine at Mount Sinai, New York, NY USA; ^2^ James J. Peters Veterans Affairs Medical Center, Bronx, NY USA; ^3^ James J. Peters Department of Veterans Affairs Medical Center, New York, NY USA; ^4^ Icahn School of Medicine at Mount Sinnai, New York, NY USA

## Abstract

**Background:**

Meta‐analysis studies provided strong correlations between individuals’ histories of early‐life depression induced by stress and their risk of developing Alzheimer’s disease (AD) [Ownby RL et al., 2006]. While psychological stress is crucial for survival, it is unknown if maladaptive stress response may function as a risk factor in the onset and progression of AD. In this study using a murine model of AD, we tested the hypothesis that susceptibility to stress may promote cognitive deterioration and AD neuropathology.

**Method:**

In this study 8‐week‐old WT and 5xFAD mice underwent a social defeat stress (RSDS) paradigm to identify differential susceptibility to stress as assessed anxiety or depression like behaviors for 2 weeks [Wang J et al., 2018], followed by novel object recognition test assessed by cognitive functions. CyTOF was used to profile peripheral immune homeostasis and unbiased RNA sequencing of hippocampal formation to assess association with changes in gene expression analyzed by CIBERSORTx deconvolution and IPA‐analysis.

**Result:**

Following RSDS exposure, we found that 61.2% of 5xFAD mice showed susceptibility and only 38.8% of total 5xFAD mice revealed resilience to psychological stress. Interestingly the ratio of susceptibility was almost opposite in WT control littermates, as reflected by only 39.4% of susceptible mice. This suggests 5xFAD mice are more susceptible to psychological stress relative to WT control littermates at 11‐weeksage. Most importantly, within 5xFAD‐stressed mouse group, we found that only the susceptible‐5xFAD mice, but not the resilient‐5XFAD mice, exhibited acceleration of cognitive impairments relative to unstressed‐5xFAD at this pre‐pathological stage of AD. There was no statistically significant difference in cognitive impairment among susceptible, resilient or non‐stressed WT control littermates. To understand potential underlying mechanisms, we performed CyTOF to identify alteration of certain blood immune cell subtypes of susceptible‐5xFAD mice compared to resilient‐5xFAD mice. Interestingly, using CIBERSORTx deconvolution and IPA‐analysis of hippocampal formation RNA seq from susceptible‐5xFAD mice which are cognitive impaired revealed significant altered expression of genes associated of synaptic plasticity relative to resilient‐5xFAD mice.

**Conclusion:**

Our study raises warnings for more investigation directed toward understanding of heterogeneity of responses when AD phenotype mice are assessed for behavior and molecular studies.

